# Effects of human milk odor stimulation on feeding in premature infants: a systematic review and meta-analysis

**DOI:** 10.1038/s41598-024-59175-4

**Published:** 2024-04-18

**Authors:** Yangyang Qin, Shu Liu, Yanming Yang, Yuan Zhong, Danshi Hao, Han Han

**Affiliations:** 1 Nursing Department, The First Affiliated Hospital of Henan University of Chinese Medicine, No. 19 Renmin Road, Jinshui District, Zhengzhou, 450099 He Nan China; 2 Henan University of Chinese Medicine, No. 156 Jinshui East Road, Jinshui District, Zhengzhou, China; 3 Obstetrics and Gynecology Department, The First Affiliated Hospital of Henan University of Chinese Medicine, No. 19 Renmin Road, Jinshui District, Zhengzhou, 450099 He Nan China

**Keywords:** Human milk odor stimulation, Premature infants, Meta-analysis, Feeding, Evidence-based nursing, Health care, Medical research

## Abstract

Previous studies suggested odor stimulation may influence feeding of premature neonates. Therefore, this systematic review and meta-analysis of randomized controlled trials was conducted to assess the effect of human milk odor stimulation on feeding of premature infants. All randomized controlled trials related to human milk odor stimulation on feeding in premature infants published in PubMed, Cochrane, Library, Medline, Embase, Web of science databases and Chinese biomedical literature databases, China National Knowledge Infrastructure, China Science and Technology Journal Database (VIP) and Wanfang Chinese databases were searched, and The Cochrane Handbook 5.1.0 was used to evaluate the quality and authenticity of the literature. Relevant information of the included studies was extracted and summarized, and the evaluation indexes were analyzed using ReviewManager5.3. The retrieval time was from the establishment of the database to July 28, 2022.12 articles were assessed for eligibility, and six randomized controlled studies were eventually included in the meta-analysis (PRISMA). A total of 6 randomized controlled studies with 763 patients were finally included in the study, and the quality evaluation of literatures were all grade B. Human milk odor stimulation reduced the transition time to oral feeding in premature infants [SMD = − 0.48, 95% CI (− 0.69, − 0.27), Z = 4.54, *P* < 0.00001] and shortened the duration of parenteral nutrition [MD = − 1.01, 95% CI (− 1.70, − 0.32), Z = 2.88, *P* = 0.004]. However, it did not change the length of hospitalization for premature infants [MD = − 0.03, 95% CI (− 0.41, 0.35), Z = 0.17, *P* = 0.86]. The implementation of human milk odor stimulation can reduce the transition time to oral feeding and the duration of parenteral nutrition in premature infants, but further studies are needed to determine whether it can reduce the length of hospital stay in premature infants. More high-quality, large-sample studies are needed to investigate the effect of human milk odor stimulation on the feeding process and other outcomes in premature infants.

## Introduction

Premature infants are babies born alive before 37 weeks of pregnancy are completed. According to the relevant World Health Organization (WHO) report, 15 million premature infants are born every year in the world^1^. Preterm birth is an important public health issue, as it is associated to a high burden of mortality and morbidities^2^. Premature infants are at high risk for aspiration due to poor coordination of sucking and swallowing^3^. Thus, they usually need tube feeding for nutritional needs with a gradual transition to oral feeding. Premature infants need to start oral feeding at the youngest possible age to improve survival and recovery^4^.

It has been shown^5–7^ that fetal olfactory receptors begin to appear in the 8th week of pregnancy, ciliated olfactory receptors mature in the 24th week, and the nasopharyngeal epithelium can express olfactory marker proteins in the 28th week. Premature infants, just like full-term infants, possess a more advanced olfactory system at birth, enabling them to detect, selectively process, retain, and recall odor information. They are able to distinguish between different odors, including those of human milk, even without history of postpartum exposure to such odors^8,9^. Olfactory stimulus refers to an environmental stimulus that uses a familiar odor or aromatic odor and is transmitted to the cerebral cortex through olfactory receptors and olfactory nerves to produce an olfactory response. In recent years, an increasing number of studies have used olfactory stimulation as a non-drug intervention to improve the effects of feeding. For example, human milk odor stimulation has a sedative effect on neonates^10,11^ and relieve the pain^12^ caused by venipuncture. The milk odor can also prevent apnea^13^ and improve oxygen saturation^14^ in premature infants.

Nutritional status parameters, including body weight and oral feeding, are key in determining whether the premature infants can be discharged in time. At present, many reports on the application of human milk odor stimulation to improve the nutritional status of premature infants, but there are differences in the research results and a lack of comprehensive evaluation. Therefore, this systematic review and meta-analysis was conducted to comprehensively evaluate the effect of human milk odor stimulation, and to provide updated evidence for the development of nursing measures in clinical practice.

## Methods

This study was conducted in conformity to the Preferred Reporting Items for Systematic Reviews and Meta-Analyses (PRISMA)^15^.

### Search strategy

Literature search was conducted from the establishment of the database to July 2022. We searched PubMed/Medline, Cochrane, Library, Embase, Web of science**,** Chinese biomedical literature databases, China National Knowledge Infrastructure (CNKI), China Science and Technology Journal Database (VIP**),** and Wanfang Data Knowledge Service Platform to retrieve published studies on the human milk odor stimulation as an intervention to improve nutritional status in premature infants. Keyword selection and search included both medical subject headings (MeSH) and life science term indexes (EMBASE TREE; EMTREE). The relevant retrieval strategy was as follows: (“Infant newborn” OR “infant” OR “newborn” OR “neonate”) AND (“Feeding” OR “nutrition” OR “feed” OR “nourishment” OR “pabulum”) AND (“Olfactory stimulation” OR “breast milk stimulation” OR “olfactory” OR “human milk” OR “breast milk” OR “odorant” OR “odor” OR “odour” OR “smell”).

### Inclusion criteria

Study characteristics used as criteria for eligibility are as follows: (1) Premature infants born at less than 37 weeks gestation who are receiving tube feeding and/or parenteral nutrition; (2) randomized controlled trials; (3) both groups of premature infants were given tube feeding and/or parenteral nutrition, with interventions involving the application of human milk odor stimulation in the intervention group and routine care in the control group; (4) evaluation metrics included transition time to oral feeding, length of stay, duration of parenteral nutrition, and/or body weight; (5) English or Chinese.

### Exclusion criteria

(1) Duplicate articles; (2) Preclinical study, meta-analysis, case reports, reviews, guidelines; (3) Valid ending data unable to be extracted or calculated; (4) Full text of the study is not available; (5) The quality evaluation is grade C.

### Data extraction

Two authors (YW and AP) carried out the data extraction process independently. Any disagreement was resolved with a senior researcher (CS) through discussion and consensus. Extracted contents were listed as follows: (1) Basic information of the included articles (title, the first author’s name, year of publication, geographic locations, the quality of the studies). (2) Baseline characteristics of the subjects in the eligible literature. (3) Detail of interventions or exposure factors. (4) The outcome indicators and outcome measures of interest (MD and SMD with the corresponding 95% CI).

### Quality assessment

The quality of the selected studies was evaluated by two investigators using a revised tool for assessing risk of bias in Review Manager software. According to the Cochrane intervention research system evaluation manual 5.1.0, the document authenticity evaluation standard is carried out^16^. It mainly includes five aspects of bias (selection bias, performance bias, detection bias, attrition bias, reporting bias), six evaluation items: the generation method of random sequence, the concealment of random scheme allocation, the blind method of subjects and interventions, the blind method of outcome evaluators, the integrity of outcome data (loss of follow-up), and the possibility of selective reporting of research results. The single evaluation item is divided into three grades: (1) “low risk of bias” when a low risk of bias was determined for all domains, (2) “high risk of bias” when high risk of bias was reached for at least one domain or the study judgment included some concerns in multiple domains, and (3) unclear risk of bias^17^. The final quality evaluation grades of the literature are Grade A, grade B and grade C.

### Statistical analysis

The main statistical software used in this study was ReviewManager5.3; Cochrane library) software. Measures such as length of hospital stay, duration of transitional oral feeding, and duration of parenteral nutrition use were statistically analyzed using the mean ± standard deviation and 95% CI. Standardized conversions could be performed with different measurement instruments to calculate MD/SMD values and 95% CI. The heterogeneity of included studies was examined by the *I*^*2*^ index. If the test showed a high level of heterogeneity (*I*^*2*^ > 50%), a random effect model was used, otherwise a fixed-effect model (*I*^*2*^ < 50%) was used^18^. Sensitivity analysis was also performed to investigate the potential interference to the pooled effect size^19^. Statistical significance was set at* P* < 0 0.05.

### Ethics approval and consent to participate

This is a systematic review, no ethics review.

## Results

### Literature search results

**Figure 1:**
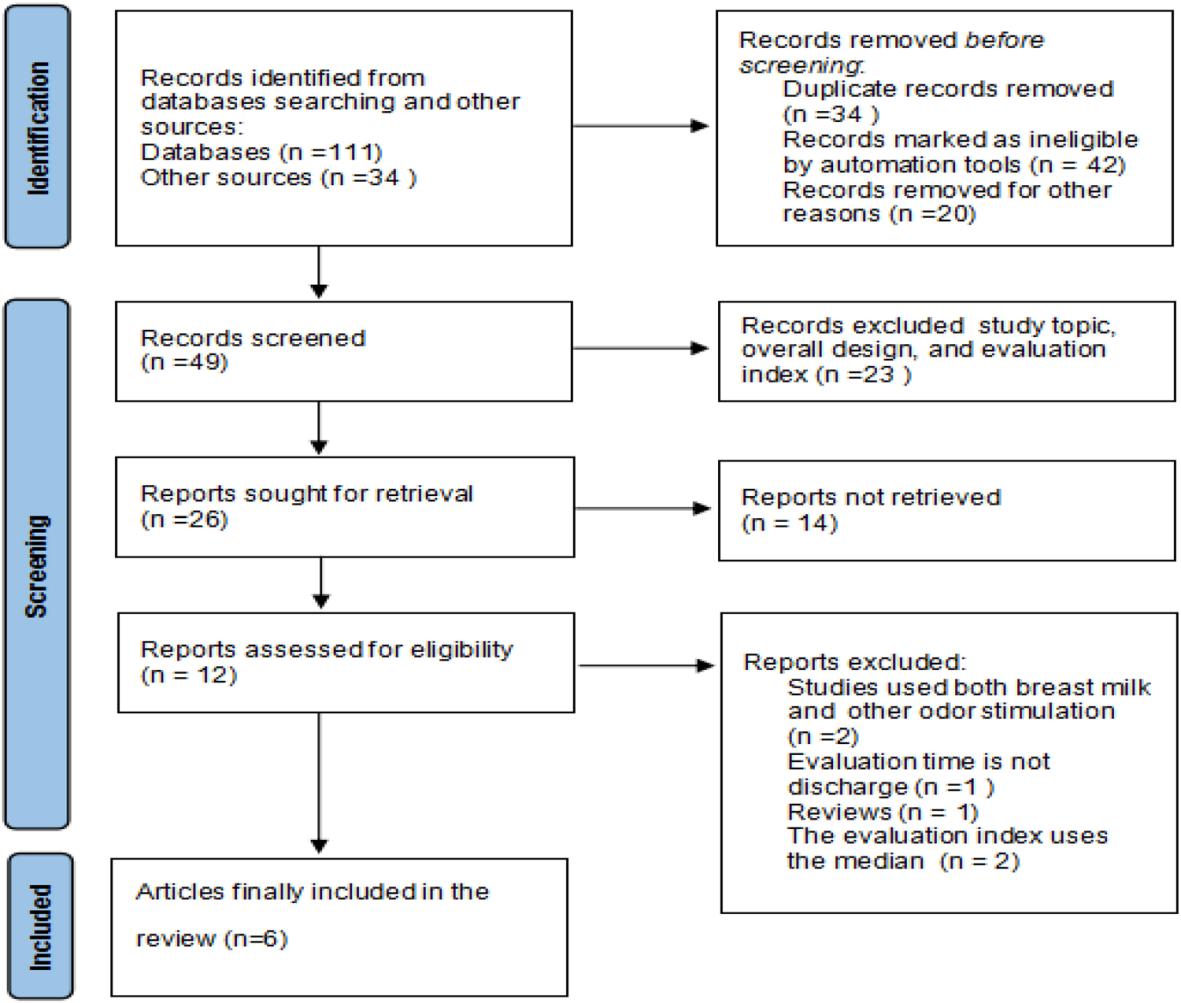
PRISMA flow diagram.

Initially, 322 literatures related to olfactory stimulation applied to premature infant were searched until July 28, 2022, of which 145 literatures related to human milk odor feeding of premature infants were screened. After excluding duplicate publications and those without full texts, 49 studies remained for full text screening. Reading through the full text, 26 articles were finally retained after excluding the inconsistent literature from the three aspects of study topic, overall design, and evaluation index. Then 12 articles were assessed for eligibility, and six randomized controlled studies were eventually included in the meta-analysis^20–25^ (Fig. 1). General information and characteristics of the included literature are detailed (Table 1).

**Table 1:** Characteristics of the included studies (n = 6).

Author year country	Number of cases Experimental/control	Interventions	Sample characteristics	Evaluating indicator
Yildiz et al 2011 Turkey	40/40	Intervention group: 1. Mother’s own milk sterile pad placed approximately 2 cm from the infant's nose at the beginning of the feeding; 2. The odor stimulation of mother’s own milk removed after the feeding was completed. A heating device was used to heat mother’s own milk to a temperature similar to body temperature so that the odor of mother’s own milk would not disappear Control group: no study intervention was performed on premature infants in the control group, except for routine tube feeding Intervention frequency and duration: performed 3 times a day; intervention until the infant gradually started oral feeding. Subjects in both study groups were followed up to hospital discharge	Premature infants born at a gestational age 28–34 weeks, with a birth weight close to 1000 g	1. Transition to full oral feeding 2. Height/weight at discharge 3. Hospitalization time
Knodagholi et al 2018 Iran	16/16	Intervention group:1. Cotton pads impregnated with the infant's mother's breast milk were used and fixed about 2–3 cm near the infant's nose. 2. Mother's breast milk supply was prepared daily by keeping it in the refrigerator in a milk storage bag and then heated with a breast milk heater that reaches body temperature to protect the natural smell of the mother's breast milk Control group: the control group was similar to the intervention group, the cotton pads were not impregnated with any substance and for the same duration and frequency Intervention frequency and duration: performed 5 min before the tube feeding feeding, 3 times a day for 10 consecutive days. Subjects in both study groups were followed up until discharge	Premature infants born at a gestational age of 28–32 weeks, with a minimum birth weight of 1000 g	1. Transition to full oral feeding 2. Height/weight at discharge 3. Hospitalization time
Lee et al 2019 Korea	12/16	Intervention group: 1. 5 ml of mother’s breast milk was dispensed at a time in sterile storage boxes and frozen in a refrigerator in the neonatal intensive care room; 2. 5 ml of frozen mother’s breast milk used to stimulate breast milk sniffing was thawed in a warm slurry bank at 60°C; 3. 5 ml of mother’s breast milk heated at 60°C was moistened with 10 × 10 cm sterile gauze and placed on the upper right side 2 cm from the nostrils of premature infants wearing disposable plastic gloves Control group: data were collected retrospectively in the control group. Indicators related to routine feeding without taking any olfactory stimulation were collected Intervention frequency and duration: mother’s breast milk olfactory stimulation was provided twice a day and three times a week for 15 days. Subjects in both study groups were followed up until discharge	Premature infants born at a gestational age of 28–32 weeks; the study was conducted on appropriate weight between 10 and 90 percentile of the gestational age curve	1. Transition to full oral feeding 2. Height/weight at discharge
Küçük et al 2011 Turkey	30/32	Intervention group: 1. Use a milking machine to extract mother’s breast milk from the mother of the premature infant. 2. Pour 5 ml of mother’s breast milk into a sterile sponge and place it 5 cm away from the infant Control group: received routine care. This included treatment, feeding. No other nursing interventions were applied Intervention frequency and duration: 1 exposure to the odor of mother’s breast milk once a day for 3 h. Intervention until the infant started oral feeding. Premature infants in the control and experimental groups were followed up simultaneously until discharge	Premature infants born at 30–34 weeks of gestational age, with a minimum birth weight of 1000 g	1. Transition to full oral feeding 2. Height/weight at discharge
Le et al 2021 China	89/76	Intervention group: 1. On the basis of conventional tube feeding, the premature infant was given olfactory stimulation 5 min before tube feeding. 2. The collected mother’s breast milk was pasteurized and refrigerated for spare, and rewarmed to 40 °C before feeding. 3. 5 ml of mother’s breast milk drops were pumped in sterile gauze (size: 5 cm × 5 cm) using a syringe, and the nurse wore sterile gloves and then held the gauze about 2 cm above the nostrils of the premature infant for about 2 min Control group: after the gastric tube was placed according to the specialized nursing operation protocol, tube feeding was performed, giving priority to mother’s breast milk, and when mother’s breast milk was insufficient, special formula for premature infants was used for feeding Intervention frequency and duration: 3 times a day, in the morning, at noon and in the afternoon before tube feeding, and stopped when the gastric tube was removed in premature infants. Subjects in both study groups were followed up until hospital discharge	Premature infants born at gestational age 30–36^+6^ weeks, birth weight 1000–2000 g	1. Transition to full oral feeding 2. Parenteral nutrition time 3 .Hospitalization time
Berker et al 2021 New Zealand	196/200	Intervention group: provide the odor and taste of breast milk (own breast milk or pasteurized donor breast milk) before each test tube feeding. A gauze swab soaked in breast milk was placed near the infant's nostrils to provide the odor Control group: infants received routine care and were not exposed to the odor of milk Intervention frequency and duration: the intervention was performed at each feeding and intervened until the premature infant was 36 weeks gestational age	Premature infants born at less than 29 weeks and/or with a birth weight of less than 1250 g	1. Parenteral nutrition time 2. Height/weight at discharge 3. Hospitalization time

### Quality assessment of the selected studies

**Figure 2:**
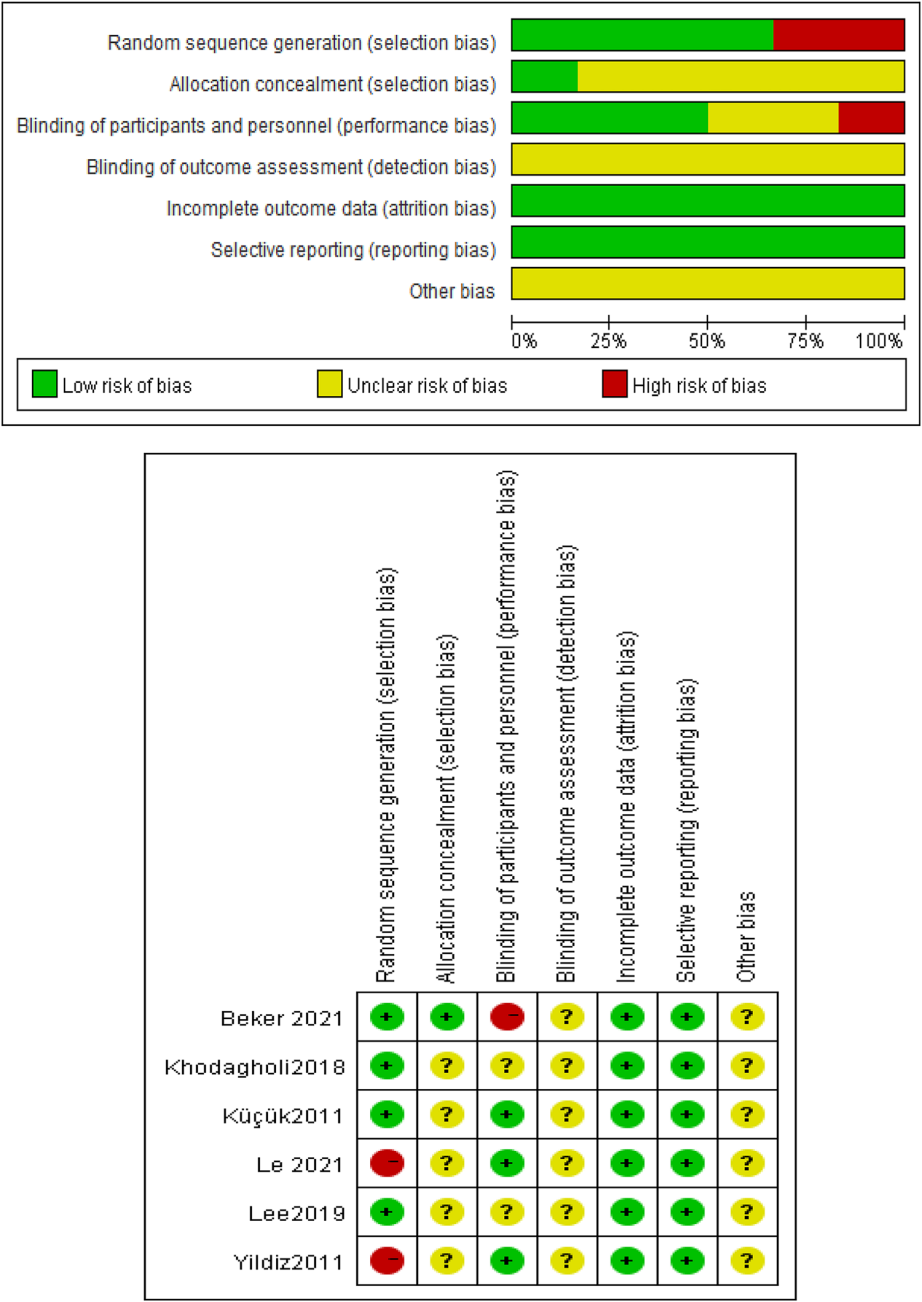
Risk of bias assessment.

The qualities of the six included literatures were evaluated as Grade B (partially meeting all criteria). Most of the studies failed to demonstrate the concealment of random scheme allocation and the blind method of outcome evaluators (Fig. 2, Table 2).

**Table 2:** Results of quality evaluation of included studies (n = 6).

Included studies	Yildiz et al	Khodaghol et al	Lee et al	Küçük et al	Le et al	Beker et al
The generation method of random sequence	High risk	Low risk	Low risk	Low risk	High risk	Low risk
The concealment of random scheme allocation	Unclear	Unclear	Unclear	Unclear	Unclear	Low risk
The blind method of subjects and interventions	Low risk	Unclear	Unclear	Low risk	Low risk	High risk
The blind method of outcome evaluators	Unclear	Unclear	Unclear	Unclear	Unclear	Unclear
The integrity of outcome data	Low risk	Low risk	Low risk	Low risk	Low risk	Low risk
The possibility of selective reporting of research results	Low risk	Low risk	Low risk	Low risk	Low risk	Low risk
Other bias	Unclear	Unclear	Unclear	Unclear	Unclear	Unclear
Quality grade (grade)	B	B	B	B	B	B

### Effects of human milk odor stimulation on the transition time of oral feeding in premature infants

**Figure 3:**
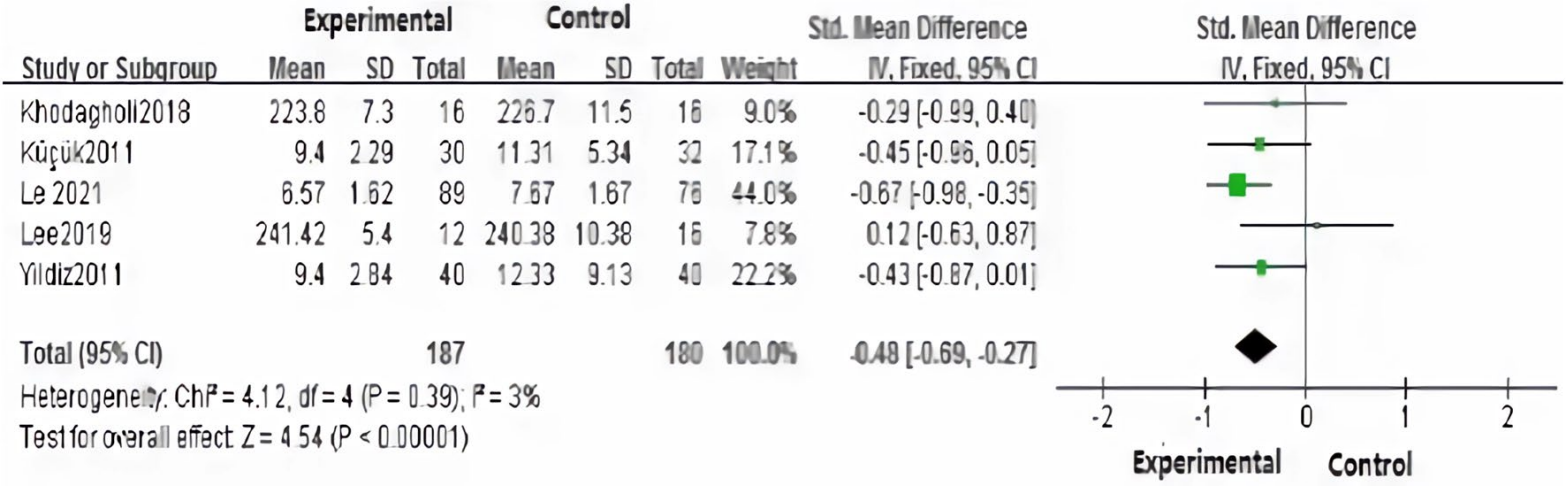
Effects of human milk odor stimulation on the transition time of oral feeding for premature infants.

Five studies^20–24^ evaluated the transition time of oral feeding for premature infants. Due to the small heterogeneity among studies (*P* = 0.39, *I*^*2*^ = 3%), fixed-effect model analysis was conducted. The result showed that the transition time of oral feeding for premature infants in the intervention group was statistically significantly shorter than that in the routine care group,the statistical unit of the outcomes were days [SMD = − 0.48, 95% CI (− 0.69, − 0.27), Z = 4.54, *P* < 0.00001] (Fig. 3).

### Effects of human milk odor stimulation on duration of parenteral nutrition in premature infants

**Figure 4:**
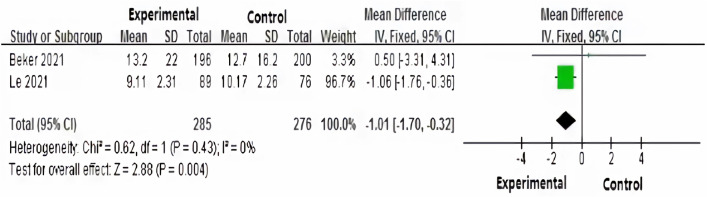
Effect of human milk odor stimulation on duration of parenteral nutrition for the premature infants.

Two studies^24,25^evaluated the duration of parenteral nutrition of premature infants. Due to the small heterogeneity among studies (*P* = 0.43, *I*^*2*^ = 0%), the fixed effect model was used to analyze the duration of parenteral nutrition. The result indicated a statistically significantly shorter duration of parenteral nutrition support in the intervention group than that in the routine care group, the statistical unit of the outcomes were days [MD = − 1.01, 95% CI (− 1.70, − 0.32), Z = 2.88, *P* = 0.004] (Fig. 4).

### Effects of human milk odor stimulation on the length of hospital stay for premature infants

**Figure 5:**
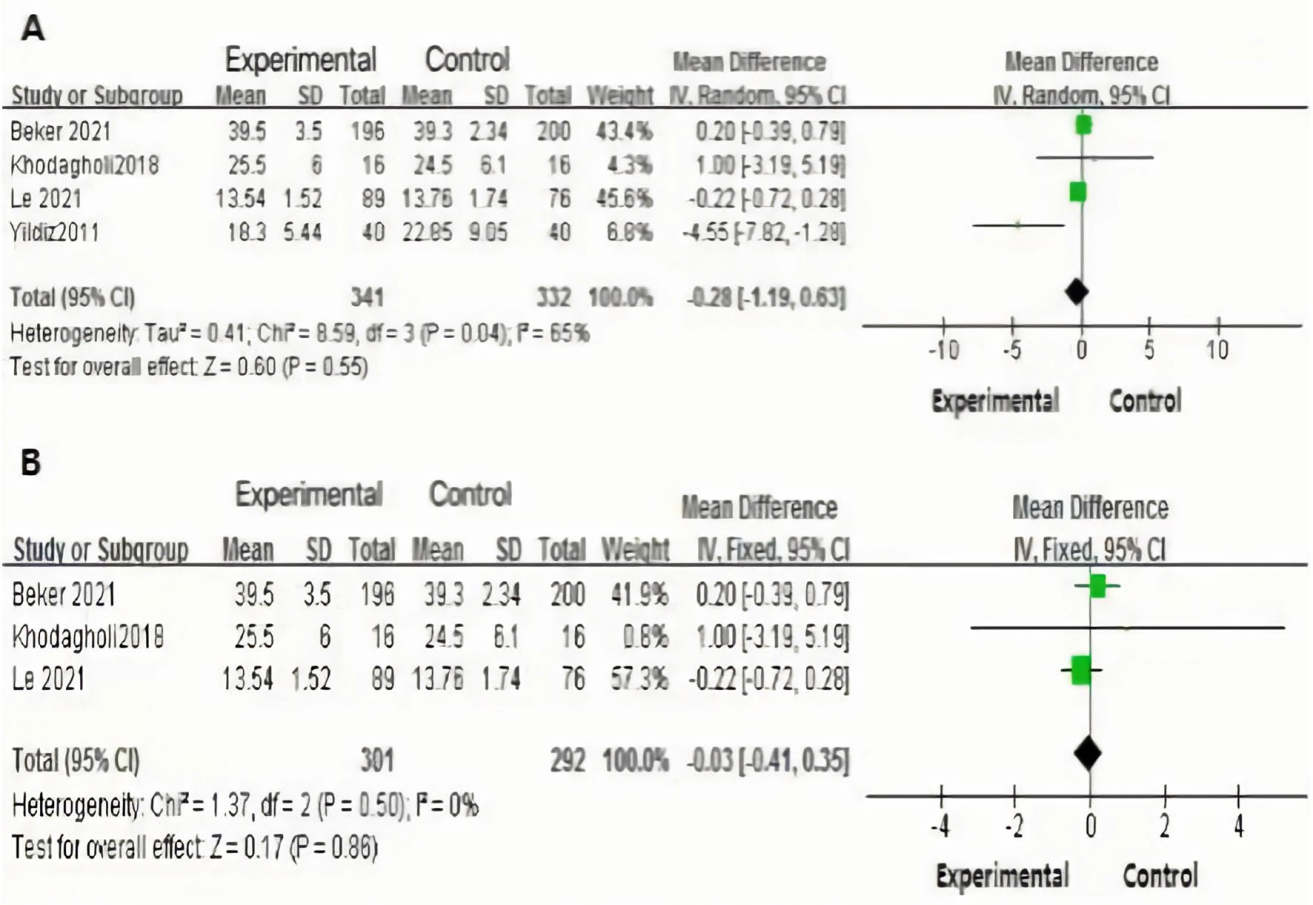
(**A**) Effects of human milk odor stimulation on the hospitalization time for premature infants before removing Yildiz’s study; (**B**) Effects of human milk odor stimulation on the hospitalization time for premature infants after removing Yildiz’s study.

Four^20,21,24,25^ studies explored the impact of human milk odor stimulation intervention on the length of hospital stay. Random-effect model was applied given the high heterogeneity (*P* = 0.04, *I*^*2*^ = 65%), which found no statistically significant difference between the intervention group and routine nursing care group [MD = − 0.28, 95% CI (− 1.19, 0.63), Z = 0.06, *P* = 0.55] (Fig. 5A). To explore the source of heterogeneity, sensitivity analysis was conducted by omitting one study at a time, which found that the study conducted by Yildiz could be have a significant impact the heterogeneity^20^. After removing this study^20^, heterogeneity was dramatically reduced (*P* = 0.50, *I*^*2*^ = 0%), and fixed effect model was used to assess the effect of human milk odor stimulation intervention on the length of hospital stay. The result still showed no statistically significant difference between the two groups, the statistical unit of the outcomes were days [MD = − 0.03, 95% CI (− 0.41, 0.35), Z = 0.17, *P* = 0.86] (Fig. 5B).

## Discussion

The systematic review and meta-analysis of six randomized controlled studies found improved outcomes of premature infants associated with human milk odor stimulation. Premature infants are difficult to be fed through bottles by mouth due to underdeveloped oral motor function and uncoordinated sucking, swallowing and respiratory movements, which usually require formula or human milk delivered through a gastric tube^26^. In addition, oral exercise by oral intake contributes to weight gain and neurological development and accelerate their recovery process, while non-oral feeding deprives premature infants of oral exercise^27–29^. Moreover, prolonged tube feeding affects the oral motor skills of the child, leading to reduced respiratory coordination, late sensory problems, and malnutrition^30^. Malnutrition leads to lack of stable weight gain, prolonged hospitalization, and even neurological deficits and readmission^31^. In contrast, adequate nutrition, maintenance of weight gain, and physiological stability play crucial roles in the successful recovery of premature infants from hospitalization^22^. Therefore, the transition from parenteral or tube feeding to complete oral feeding will contribute significantly to sufficient nutrition and prompt recovery of premature infants. The results of the pooled analyses in the present study showed that human milk odor stimulation was able to reduce the time required for transition to normal oral feeding in premature infants. It is well known that normal oral feeding (sucking, swallowing and respiratory coordination) is an early sign of neuromotor integrity in premature infants and an important indication for hospital discharge^28^.

Premature infants admitted to the newborn intensive care unit (NICU) for further treatment and care after birth often require controlled number, frequency, and volume of feedings. Therefore, these newborns may lack adequate stimulation and sensory experiences related to feeding, such as hunger, fullness, taste, and smell^32^. Olfactory and gustatory stimulation alone or in combination can reduce gastrointestinal-related adverse reactions and effectively improve the nutritional status of premature infants by activating complex pathways and triggering cephalic responses, which increase intestinal motility, digestive enzyme secretion, and hormone release^33–35^. Therefore, the application of human milk odor stimulation plays a vital role in promoting the recovery process of premature infants.

Although human milk odor stimulation was associated with reduced transition time to oral feeding and short duration of parental feeding, it did not change the length of hospitalization. Hospitalization in premature infants is affected by a variety of confounding factors, such as body weight, gestational age, the occurrence of complications, family economic status, and medical environment factors. Relevant reports found that very low birth weight infants had significantly longer hospital stays. Moreover, it also revealed that the smaller the gestational age was, the more likely it would be to have complications such as infection, cerebral hemorrhage, and pulmonary hemorrhage, thus prolonging the length of hospital stay^36^. Due to differences in medical and economic levels in different countries, there will also be inconsistencies in the length of hospitalization for premature infants^37^.

It is noteworthy that the study by Küçük^23^, Beker^25^, Khodagholi^21^, and Yildiz^20^ reported the weight of premature infants at discharge, and the mean weight at discharge of the control group of these infants in the four studies was 1933.10 ± 90.50 g, 2913 ± 577 g, 1588.1 ± 84.4 g, and 1922.25 ± 230.82 g, respectively. In contrast, the mean weight of the intervention group at discharge were 1908.00 ± 87.86 g, 2986 ± 672 g, 1565.6 ± 93.6 g, and 1893.50 ± 189.04, respectively. However, the difference of the discharge weight between the intervention group and the control group in each study was not statistically significant. Because the initial weight of the premature infants at admission was different between the control group and the intervention group, a simple comparison of body weight at discharge did not yield enough information. Therefore, the effects of the weight of premature infants were not included and observed.

Several inherent limitations need to be noticed when interpreting the results of this meta-analysis. First, the number of studies included was small with overall small sample size. Second, all included studies had a quality rating of B, which may have had an impact on the evaluation results. Third, other parameters, such as weight gain in premature infants, were not available and their effects were not assessed.

## Conclusion

This systematic review and meta-analysis found that human milk odor could reduce the transition time to oral feeding and duration of parenteral nutrition for premature infants, suggesting a cheap, effective, and easily accessible method to improve the overall outcomes of premature infants. However, the findings are limited by the number and quality of included studies, therefore, more well-designed studies are still needed to verify our findings.

## Data Availability

The data that support the findings of this study are available from the corresponding author upon reasonable request.
